# Characterization of the intestinal microbiota of the sea cucumber *Holothuria glaberrima*

**DOI:** 10.1371/journal.pone.0208011

**Published:** 2019-01-30

**Authors:** María Pagán-Jiménez, Jean F. Ruiz-Calderón, María G. Dominguez-Bello, José E. García-Arrarás

**Affiliations:** Biology Department, University of Puerto Rico, Río Piedras Campus, San Juan, Puerto Rico; Institute of Subtropical Agriculture, Chinese Academy of Sciences, CHINA

## Abstract

High-throughput 16S rRNA gene sequencing has been used to identify the intestinal microbiota of many animal species, but that of marine invertebrate organisms remains largely unknown. There are only a few high-throughput sequencing studies on the intestinal microbiota of echinoderms (non-vertebrate Deuterostomes). Here we describe the intestinal microbiota of the sea cucumber *Holothuria glaberrima*, an echinoderm, well-known for its remarkable power of regeneration. We characterized the microbiota from the anterior descending intestine, the medial intestine (these two comprise the small intestine) and the posterior descending intestine (or large intestine), using pyrosequencing to sequence the V4 region of the 16S rRNA gene. We compared animals in their natural marine environment and in sea-water aquaria. A total of 8,172 OTU’s were grouped in 10 bacterial phyla, 23 classes, 44 orders, 83 families, 127 genera and 1 group of unknown bacteria, present across the digestive tract of 10 specimens. The results showed that the anterior intestine is dominated by Proteobacteria (61%) and Bacteroidetes (22%), the medium intestine is similar but with lower Bacteroidetes (4%), and the posterior intestine was remarkably different, dominated by Firmicutes (48%) and Bacteroidetes (35%). The structure of the community changed in animals kept in aquaria, which had a general dominance of Firmicutes and Bacteroidetes, regardless the intestinal segment. Our results evidence that in the natural sea environment, there is intestinal segment differentiation in the microbiota of *H*. *glaberrima*, which is lost in artificial conditions. This is relevant for physiological studies, such as mechanisms of digestive regeneration, which might be affected by the microbiota.

## Introduction

The microbiome refers to the genome of microbial life forms inhabiting a living host, and their interactions with the host [1]. The term was first suggested by Joshua Lederberg to describe the collective genome of our indigenous microbes and to introduce the idea that a genetic view of humans should include the microbial genes [2]. They play significant roles in the metabolism of the host. Among these the most studied have been the hydrolysis of ingested molecules, the synthesis of vitamins [3] and the stimulation of the immune system [4,5]. Other microbiota studies addressed the development of obesity [6,7]), the integrity of the intestinal mucosal barrier ([8–10], the proliferation and differentiation of epithelial lineages during intestinal development [11,12]. and the activity of the enteric nervous system [13,14], changes in the host behavior [15,16–19] and the microbiome associated to diseases, such as cancer [20,21].

The current knowledge of the gastrointestinal microbiome and its benefits are mainly focused on vertebrates particularly on mammals. Among marine animals, two of the groups most studied in terms of their microbiota are sponges and corals [22–24], however there are few investigations of other marine invertebrates.

Members of the phylum Echinodermata comprise some of the most important marine invertebrates. They are found in all marine environments, from coastal to benthic and from the tropics to the polar regions. In some of these they constitute the majority of biomass present [25]. Echinoderms include five different classes: *Asteroidea* (sea stars), *Echinoidea* (sea urchins and sand dollars), *Crinoidea* (crinoids or sea lilies), *Ophiuroidea* (brittle stars) and *Holothuroidea* (sea cucumbers). Culture-dependent studies of the microbial composition in the intestine of adult holothurians (and other echinoderms) have shown that they have a great diversity of microorganisms, such as bacteria, viruses, protozoa, and fungi that colonize the intestine [26]. Studies have shown the presence of bacteria inhabiting the guts in echinoids [27,28], holothuroids [29–33], and ophuiroids [29]. Some studies have focused on the bacteria found in specific compartments of the digestive tract, particularly in the foregut [34], intestine [31, 35–37], hindgut ([34], and cecum [27]. The characterization of bacteria in the gut showed that ~50% of the isolates were related to members of the genus *Vibrio* and neighboring taxa. Other isolates, included members of the genus *Bacillus*, the alpha and gamma subclasses of the Proteobacteria, the *Cytophaga-Flavobacterium-Bacteroides* lineage, and the order *Actinomycetales* [38]. In addition, it was found that gut microbiota of two species, *A*. *japonicus* and *Holothuria leucospilota*, are involved in the breakdown of indigestible products during intestinal metabolism [39–41].

Here we studied the microbiota of the sea cucumber *Holothuria glaberrima*, and determined the differences between individuals from natural and aquarium environments. This study is important for two different reasons. First, there is limited information on echinoderm microbiotas with only one study on the microbiota of holothurians [33] and two in sea urchins [42,43]. Our study contributes information on a holothurian species from a different ecological niche. *H*. *glaberrima* lives in the coastal rocky shore feeding on organic matter brought by the continuous wave action. Particulate matter, including algae, sand, mud, organic and inorganic debris, etc. are picked by the animals tentacles and introduced into the mouth.

The second, and most important reason (from our laboratory perspective), *H*. *glaberrima* has become an important model system to study intestinal regeneration [44–46]. This study provides the fundamental information on the microbiota of this species in natural and aquarium environments, thus paving the way for future studies on the changes in bacterial compositions associated with the intestinal regeneration process.

## Materials and methods

This research deals only with invertebrate animals, thus the University of Puerto Rico IACUC waives ethical approval of research performed on invertebrates. Animals were sacrificed by sectioning the anterior part of the animal close to the oral nerve ring, which accounts for the principal nervous component.

### Sample collection

Ten adult animals were captured from their natural habitat in Playa Piñones, Puerto Rico. Permission is not required for their capture since these animals are not either endangered or protected. The coastal area where they were collected is not private property and is considered public property. Five of the animals were dissected *in situ* while the remaining five were transported to the lab and placed in seawater aquarium.

**Fig 1:**
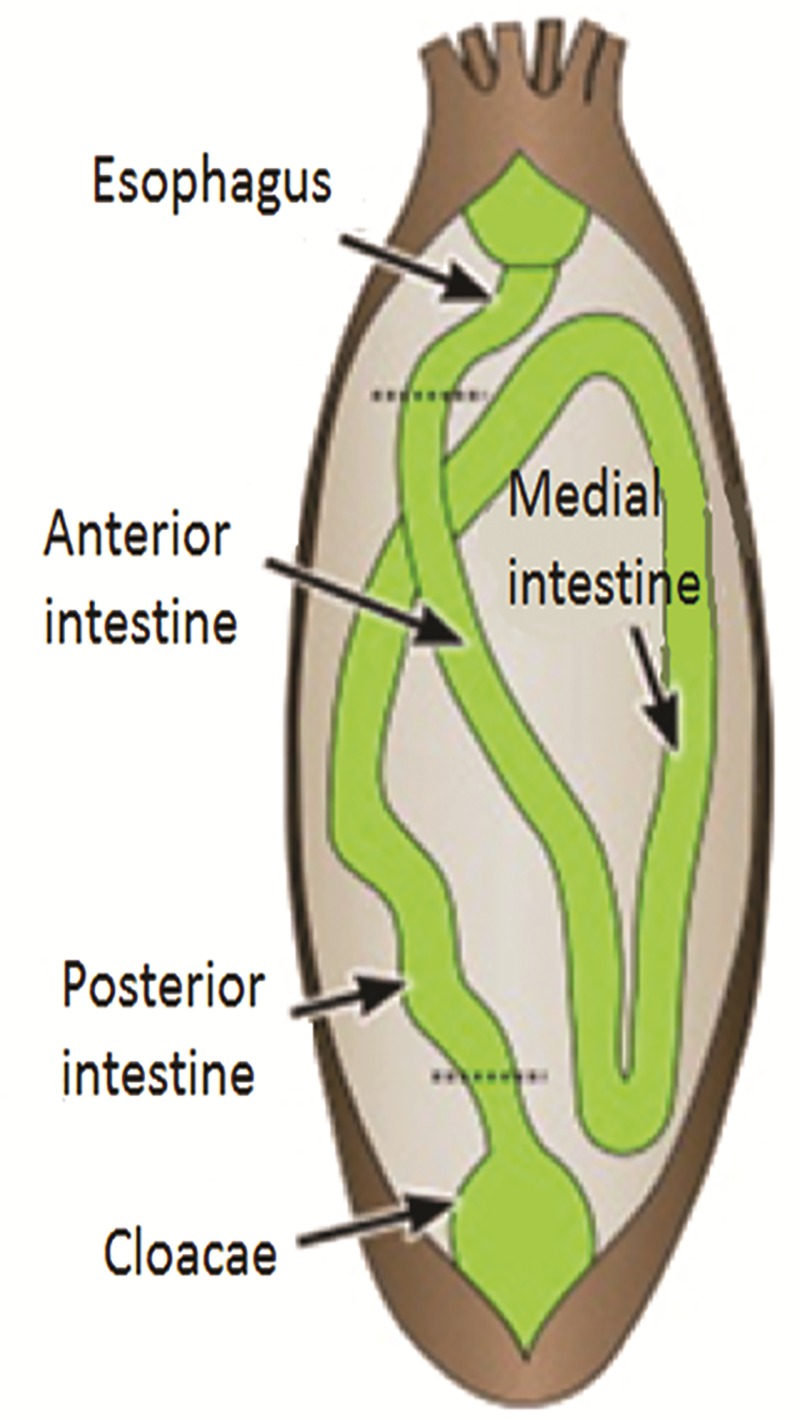
Anatomy of the digestive tract of the sea cucumber *H*. *glaberrima*. The digestive tract of *H*. *glaberrima* is formed by a continuous tube that begins at the mouth, forming a short esophagus which is attached to a long descending small intestine (anterior) and a long ascending small intestine (medial). The final segment of the tube is a descending large intestine (posterior) that ends in the cloacae. (Diagram obtained from Mashanov et al. 2012. Adapted by MPJ).

Intestines that were dissected in situ were filled with the usual sand, algae, organic matter and other debris that the animals acquired by capturing from their surroundings with their tentacles and inserting them into their esophagus. These intestines were rinsed in seawater to remove most of the content. Each intestine was divided into three segments ranging from 5 to 7 cm: the anterior segment, which extends from the esophagus to the first descending intestine; the medial segment, which encompasses the ascending small intestine; and the posterior segment, which is the second descending or large intestine that ends in the cloacae (Fig 1). Dual cotton swabs (BD Diagnostics, BD-220135, Franklin Lakes, NJ USA) were used to collect the microbial sample from the luminal epithelium of each segment, and the samples were stored in a 1.5 mL centrifuge-tube containing 200 μl of sucrose lysis buffer (20mM EDTA, 400 mM NaCl, 0.75 M Sucrose, 50 mM Tris-HCl, pH 9.0)(Suppl. Venter et al. 2011). All samples were immediately frozen in dry ice at -78°C, and were transported to the University of Puerto Rico where the DNA was extracted. In addition, two liters of seawater were transported to the lab, and used as a control to determine what microorganisms were present in the surrounding environment. To obtain the microbial sample from the seawater, the two liters of water were filtered through 11 mm sterile filter paper (Qualitative 1, Whatman Filter Paper) to remove large particles from the water. The water was again filtered through Millipore membrane filters (0.45 μm pore size), and then filtered through another Millipore membrane filter (0.22 μm pore size) to obtain the bacterial cells. The two Millipore membranes (0.45 μm and 0.22 μm) were removed from their respective filter and were transferred to a sterile 15 mL centrifuge-tube with 10 mL of sucrose lysis buffer and stored at -20°C until DNA extraction.

The five animals that were transported to the lab were kept in a sea water aquarium for 24 hours. The sea water used in the aquaria was sea water brought from the animals’ natural environment. During this time the animals eliminated most of their intestinal content via the cloaca, and once this occurred, specimens were transferred to a sea water aquarium with fresh natural sea water to minimize the amount of digestive tract material present. These animals were dissected after three days in the aquarium, and digestive tract samples were taken using the same protocol as for the *in situ* dissections described above. By keeping animals in the aquaria for 3 days we reproduced the conditions that are used in regeneration experiments [44–46]. Therefore, the acquired data, and the comparison to natural environments will be important for future experiments on the association of bacteria with regenerative events taking place in the laboratory aquaria.

### DNA extraction from intestine and water samples

DNA extraction was performed according to the manufacturer protocol (Qiagen's QIAamp DNA Mini Kit) (#51306, Valencia, CA US). For the DNA extraction of seawater bacteria, we removed the membranes and the remaining sucrose lysis buffer contained in the 15mL centrifuge-tube was centrifuged at 10,000 rpm for 30 minutes. The supernatant was discarded, and the pellet was dissolved in 1–2 mL NaCl (0.9%) to perform the DNA extraction following the same protocol as for the intestinal samples. DNA quantification was determined by absorbance measurements using a NanoDrop (1000 Spectrophotometer, Thermo Fisher Co.) device. The amount of DNA per sample varied from 2.6 mg/μl to 22.2 mg/μl. All samples were stored frozen at -20°C until used.

### Sample preparation for pyrosequencing of 16S rRNA genes

PCR for multiplexing pyrosequencing was performed using universal bacterial barcoded primers. A set of primers was designed by adding a 12-nucleotide barcode to the forward primer 515F (5’-GAGTGCCAGCMGCCGCGGTAA). The reverse primer (not barcoded) was 806R (5’-CCGGACTACHVGGGTWTCTAAT). These primers targeted the V4-V5 regions of the *16S* gene of bacteria for amplification. PCR was performed with a thermal cycler (PTC 100, Bio-Rad) under the following conditions: initial denaturation at 94°C for 3 min; 35 cycles at 94°C for 30 s, 50°C for 30 s, 72°C for 1 min; and a final extension at 72°C for 10 min. The PCR preparation consisted of 5μl of DNA, 2.5μl of barcoded primers and 10μl of Master Mix (Promega #M7502). PCR products were purified using Ultra Clean PCR Clean-Up (MoBio #12500) and were quantified with Quant-IT PicoGreen dsDNA Assay Kit (Invitrogen Cat # P11496). A mixture of PCR products was prepared and then was pyrosequenced using the Roche 454 FLX Titanium platform at the Sequencing and Genotyping Facility of University of Puerto Rico, according to the manufacturer’s instructions.

### Taxonomic assignments and species richness of pyrosequencing reads

Statistical and bioinformatic analyses of bacterial 16S amplicons were done using QIIME pipeline to process data from high-throughput 16S rRNA sequencing studies [47]. Multiplexed and trimmed sequence reads (300bp) were clustered into OTU’s (Operational Taxonomic Units) at 97% sequence identity using UCLUST to estimate richness. The alignment of the sequences was done by PyNAST against the Greengenes core set. The OTU classification was done using RDP (Ribosomal Database Project)-classifier [48]. FastTree was used for building a phylogenetic tree [47]. Prior to phylogenetic tree building, the alignment was filtered to remove positions with gaps.

### Comparison of microbial communities

Beta diversity metrics were calculated for each sample and the types of communities were compared using the taxonomic and phylogenetic assignments. UPGMA and PCoA plots were generated to visually depict the differences between the samples [47]. Beta significances were calculated as an “unweighted and weighted unifrac” which performs randomizations of sample/sequence assignments, and records the probability that one sample is phylogenetically different from the other samples, using Permutational multivariate analysis of variance (PermANOVA) test.

## Results

A total of 138,029 V4-V5 16S rRNA gene sequences (~300bp) were obtained. The sequences were binned into 8,172 OTU’s (threshold cutoff for each OTU, 97% nucleotide sequence identity using UCLUST). The OTU classification was done using the RDP-classifier and we obtained 10 bacterial phyla, 23 classes, 44 orders, 83 families and 127 genera, that were present along the sea cucumber digestive tract. In terms of microbe relative abundance, the most abundant phyla were the *Firmicutes (39*.*1%)*, *Bacteroidetes (24*.*4%)* and *Proteobacteria (23*.*8%)*, followed by the *Fusobacteria* (4.2%) and *Actinobacteria* (1.3%). Unknown bacterial phyla represented 6.5% of OTU’s.

Within the *Bacteroidetes*, the most abundant genera included *Bacteroides (2*.*5%) and Lewinella (1*.*3%);* the families *Porphyromonadaceae (8*.*9%) and Bacteroidaceae (2*.*5%);* and the order *Bacteroidales (3*.*2%)*. The most abundant groups in the *Firmicutes* were the genus *Lactobacillus (2*.*1%);* families, *Lachnospiraceae (13*.*2%)* and *Ruminococcaceae (2%)*; and order *Clostridiales (18*.*6%)*. The most abundant groups in the *Proteobacteria* were genera *Vibrio* (11.7%), *Shimia (1*.*1%) and Helicobacter (1*.*2%);* and order *Oceanospirillales (2%)*. Within *Fusobacteria* the most important group was family *Fusobacteriaceae (10%)*, and from *Actinobacteria* was the genus *Corynebacterium (7%)*.

### Bacterial distribution along the three segments of the intestine in the natural coastal environment

**Fig 2:**
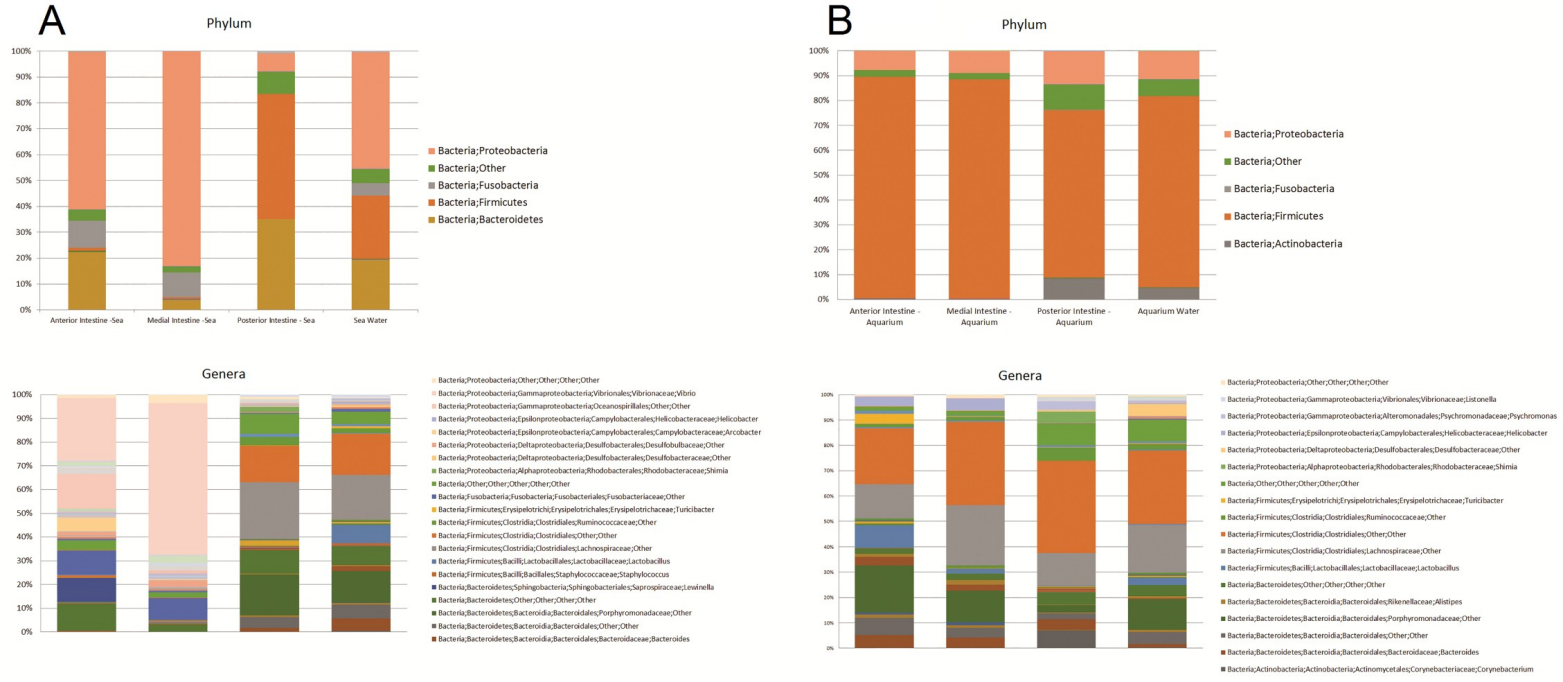
Bacterial taxa distribution in the intestinal system of *H*. *glaberrima*. (A) Phylum and Genera of intestinal bacterial OTUs of animals in the sea (natural environment). B) Phylum and Genera of intestinal bacterial OTUs of animals in seawater tanks (aquarium environment).

The microbiota found in different areas of the digestive tract of animals in their natural environment was similar at the phylum level. However, the relative representation differed remarkably. The results showed that the anterior and the medial (small) intestines are more similar between them when compared to the posterior (large) intestine. The former showed a greater proportion of *Proteobacteria* (61% in the anterior and 83% in the medial) and *Fusobacteria* (10%) and a smaller proportion of *Firmicutes* when compared to the posterior intestine (Fig 2). Notwithstanding, there were also differences between the two small intestinal segments, where the anterior intestine had a greater proportion of *Bacteroidetes* (22%), while the medial intestine only showed 4%. The posterior intestine was very different, with the most abundant phylum being the *Firmicutes* (48%), followed by *Bacteroidetes* (35%). In contrast with the anterior and medial intestine, the posterior intestine showed greatly reduced percentages of *Proteobacteria* (7%) while no *Fusobacteria* could be detected. Finally, the seawater sample reflects the taxonomy found in the three intestinal segments. *Proteobacteria* group 45%, *Firmicutes* 24%, *Bacteroidetes* 19%, and *Fusobacteria* 5% of the bacterial relative abundance. Similar to the digestive tract, a small number of bacteria (5%) could not be classified.

At more specific levels, the anterior and medial intestines are dominated by the genus *Vibrio* (26% and 64% respectively), the families *Fusobacteriaceae* (10%) and *Desulfobulbaceae* (2% and 3% respectively), and other *Bacteroidetes* (11% and 3% respectively). In addition, the anterior intestine is dominated by the order *Oceanospirillales* (15%), and the genera *Lewinella* (10%) and *Arcobacter* (6%), whereas in the medial intestine these groups appear to be displaced by the genus *Vibrio*. On the other hand, the posterior intestine is different from the other two intestinal segments and is more similar to the seawater sample, where the *Firmicutes* and *Bacteroidetes* are dominant. Among the *Firmicutes* bacteria in the posterior intestine and seawater, the most abundant groups are: the *Lachnospiraceae* family (24% and 19% respectively), the order *Clostridiales* (15% and 17% respectively), and from this order, the *Ruminococcaceae* family (4% and 2% respectively). Secondly, the *Bacteroidetes* phylum is highly represented by the family *Porphyromonadaceae* (17% and 13% respectively), the order *Bacteroidales* (5% and 6% respectively) and others *Bacteroidetes* (10% and 8% respectively). In addition, we found a low representation of other *Proteobacteria* in both samples, except for the peculiar finding that the genus *Shimia* (2%) is found only in the posterior intestine segment.

### Bacterial distribution along the three segments of the intestine in the aquarium environment

Many of the experiments performed in our laboratory require that animals be maintained in indoor seawater aquaria for prolonged periods of time. It is possible that the microbiota of animals in these conditions varies from that of animals in their normal habitats. To determine the microbiota of animals within the aquaria, we analyzed the bacterial taxonomy from intestinal samples after 3 days in the aquaria. Our results show that the microbiota of the digestive tract of animals in the aquaria was similar among the three different segments in terms of taxonomy and relative abundance. Their bacterial composition showed a large proportion of *Firmicutes* and *Bacteroidetes*. The *Proteobacteria* group is the least represented in the digestive tract of animals in the aquarium environment (Fig 2). On the other hand, we observed that the posterior intestine and aquarium water samples have representatives of *Actinobacteria*, a group of bacteria not found in the other samples.

At more specific levels all the samples have a similar taxonomic distribution. The dominant groups in the *Bacteroidetes* are the genera *Bacteroides* and *Alistipes*, and the family *Porphyromonadaceae*. For the *Firmicutes*, the dominant groups are the genera *Lactobacillus*, *Turicibacter* and *Helicobacter*; the families *Lachnospiraceae* and *Ruminococcaceae*; and the order *Clostridiales*.

On the other hand, as mentioned above, the posterior intestine has a representation of *Actinobacteria* that is dominated by the genus *Corynebacterium (7%)*. Similar to animals in their natural environment, the posterior intestine from aquarium environment is the only sample that contains the genus *Shimia* (4%).

### Beta-diversity of bacterial communities among water and intestinal samples

**Fig 3:**
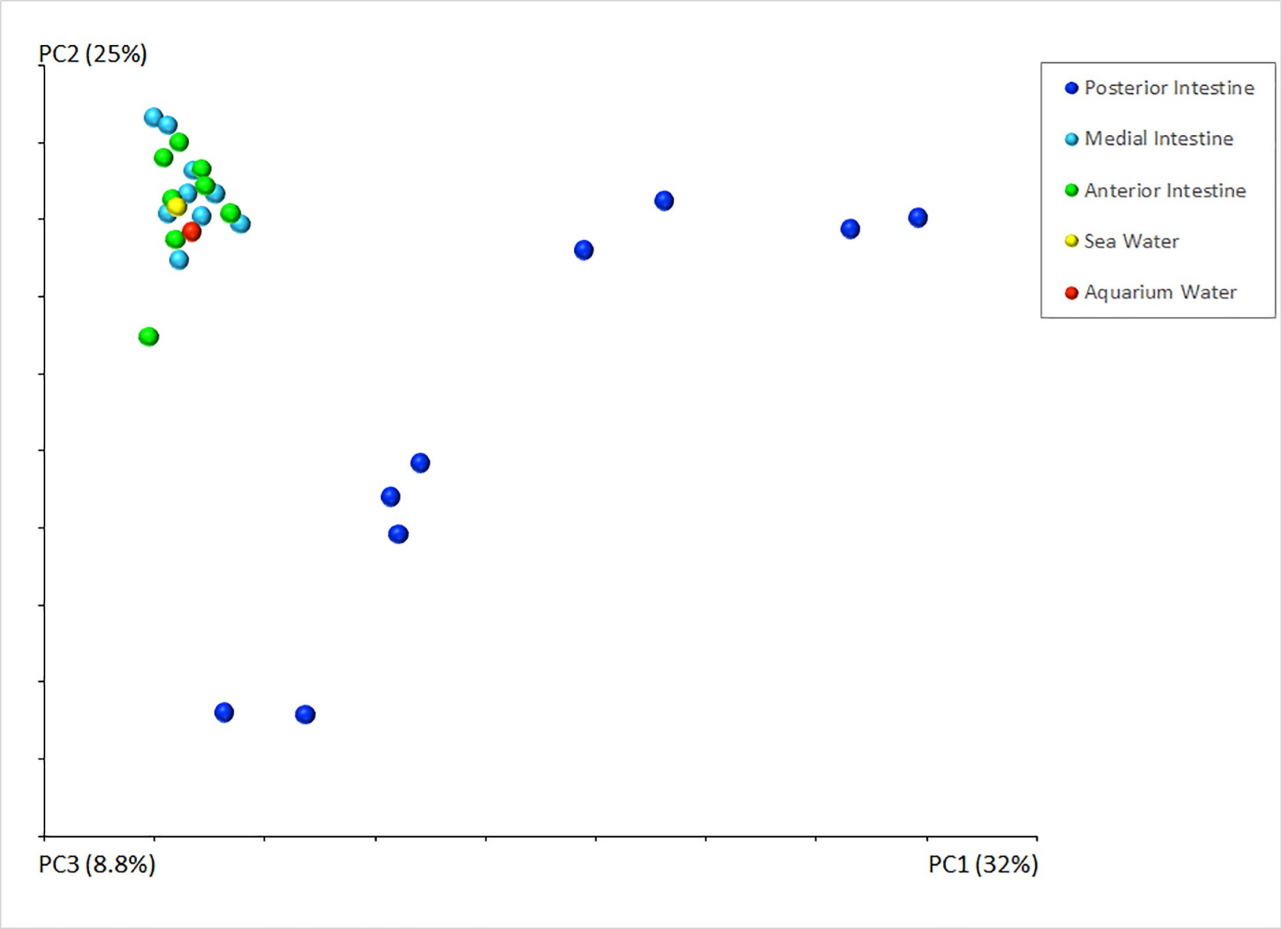
Principal Coordinate Analyses (PcoA) of bacterial communities in the intestine of *H*. *glaberrima*. Samples clustered using PcoA of weighted UniFrac distance matrices that reflect the beta-diversity of the bacterial communities. The graph shows the UniFrac distance of bacterial communities from the anterior, medial and posterior intestine of the sea cucumber *H*. *glaberrima*.

The weighted PCoA revealed that the anterior, medial and posterior intestine bacterial communities formed three significantly different clusters (*P* = 0.005). We compared the three intestinal segments, and the resulting graph showed a separation of the posterior intestine segment from both anterior and medial intestine segments (Fig 3) with a significance difference (p = 0.001). These results showed a concordance with the bacterial richness at phylogenetic levels (phylum), where the anterior and medial intestine segments shared greater similarity (No significant differences were found between anterior vs. medial and posterior intestine, or between medial vs anterior and posterior intestine. In general, both the aquarium water and the seawater samples were more similar to the anterior and medial intestine than with the posterior intestinal segment (Fig 3).

### Beta-diversity of bacterial communities between the host environments

**Fig 4:**
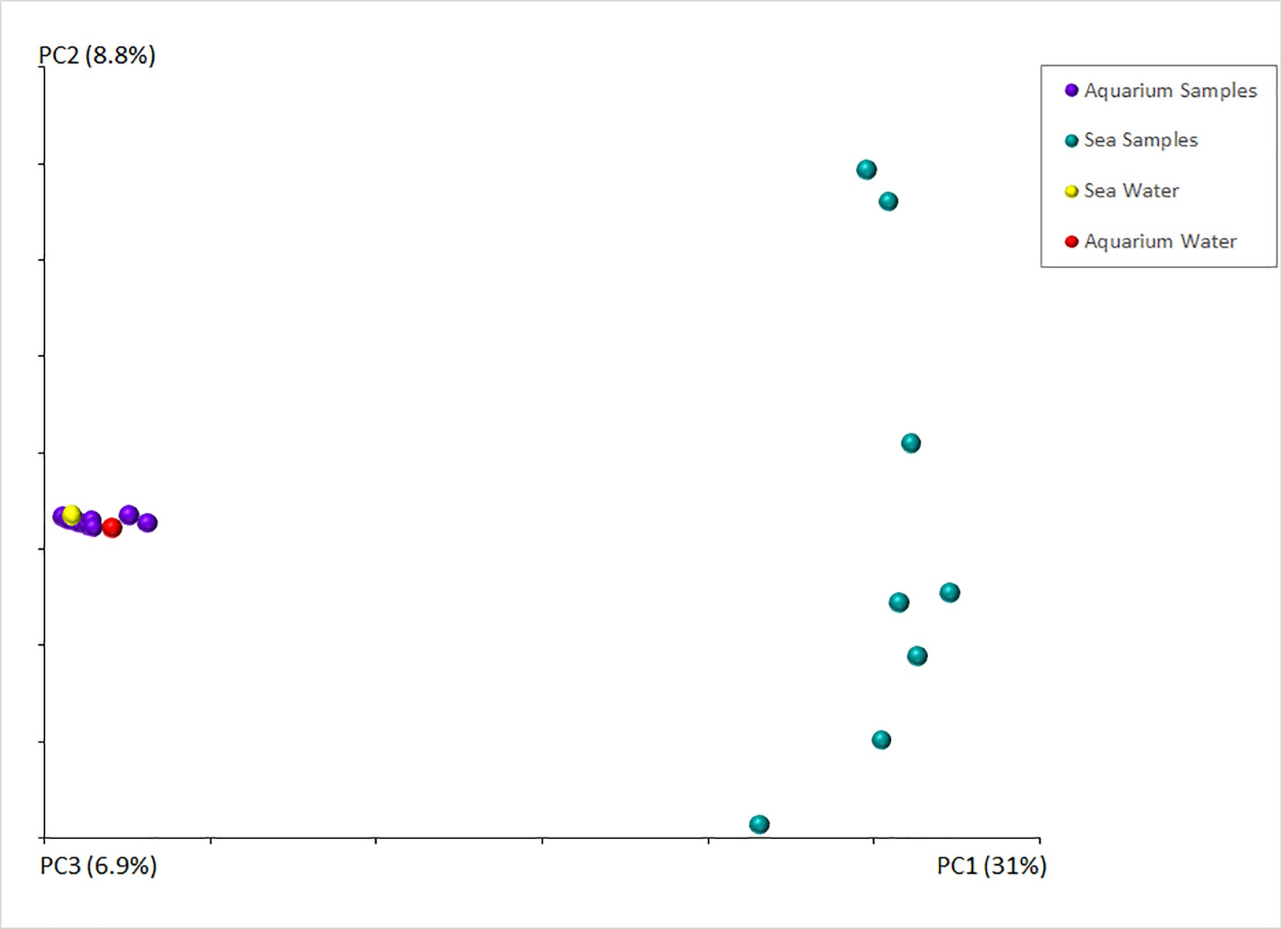
Principal Coordinate Analyses (PcoA) of bacterial communities in the intestine of *H*. *glaberrima* in two different environmental settings. The PcoA analysis reflects the beta-diversity of the bacterial communities from the various intestinal segment samples that originate from two environments; the animals collected in their natural sea environment and animals kept in seawater aquaria for 3 days.

The UniFrac metric revealed that the samples from sea and aquaria formed two significantly different clusters (P = 0.001) based on the origin of the samples (Fig 4). Therefore the bacterial compositions of the host in the two environments are significantly different. Moreover, the seawater sample clustered with the aquarium intestinal samples (Fig 4).

## Discussion

In accordance to many other studies where 16S data is used to determine microbial diversity, the number of genera identified by our study is much larger than the ~20 genera that have been identified in the digestive tract of several holothurian species using culture-dependent methods [38, 49]. This confirms the general finding that the use of “culture-dependent” methods to assess microbial diversity only detect a limited group of microorganisms; therefore, they cannot be used to define the entire microbiota within the intestine [50].

### *H*. *glaberrima* intestinal microbiota comparison with other organisms

In this study, the characterization of intestinal bacteria of *H*. *glaberrima* revealed a dominance of *Bacteroidetes*, *Firmicutes* and *Proteobacteria*. *Bacteroidetes and Firmicutes* are typical dominant members of the vertebrate gut, particularly in mammals [51, 52], where the *Bacteroidetes* phylum is highly represented by the genus *Bacteroides*, and *Firmicutes* is mostly represented by the genera *Clostridium*, *Ruminococcus*, and *Lactobacillus* [1], this representation is similar to the most abundant taxonomy found in our study. Many studies have demonstrated that bacterial members of these two phyla are important for the normal intestinal physiology and homeostasis of vertebrate host [6, 7, 53]. Thus, our findings suggest that the gastrointestinal tract of marine and terrestrial deuterostomes share common microbial groups that can influence in the gastrointestinal metabolism of the host. The phylum *Proteobacteria*, the third most abundant phyla in *H*. *glaberrima*, has been found as a common member of the gut microbiota in adult zebrafish [54]. Both animals inhabit aquatic environments and it has been shown that in oceans and aquatic environments, *Proteobacteria* is the most abundant phylum comprising 79% of the bacterial biomass in deep sea, 64% in the sea surface, and 40% in fresh water [55].

The predominance of the phylum *Proteobacteria* is consistent with previous studies of the bacterial gut composition of other marine invertebrates [56,57]. Studies in the guts of Crustacea [*Macrobrachium rosenbergii* [58]; *Palaemon paucidens* [59]; *Penaeus aztecus* [60]; Mollusca [*Donax gouldii* [61], and Echinodermata [*Echinus esculentus* [37] reveal that genera members of *Proteobacteria*, such as, *Vibrio* and *Pseudomonas*, are commonly isolated in the three invertebrates phyla [57]. *Vibrio* is the most abundant genus found in *H*. *glaberrima* and it is consistent with studies in other echinoderms such as the sea urchins *Strongylocentrotus droebachiensis* and *Tripneustes ventricosus* [28], and the ophiuroid *Ophionema sp*, that suggest that echinoderms have a high population of *Vibrio spp*. in the gut, that may serve as reservoirs for the bacteria [24]. In addition, the phylum *Proteobacteria* has been found in high abundance in culture-dependent studies of members of the Holothuroidea: *Benthodytes sp*. [29], *Stichopus japonicus* [39], *Holothuria atra* [38], *Holothuria leucospilota* [40] and *Apostichopus japonicus* [33].

### Taxonomic comparison between *H*. *glaberrima* microbiota and those of other echinoderms

Studies of the gut microbiota in echinoderms have been few, moreover high throughput sequencing studies are scarce. Using 454 pyrosequencing, Gao and colleagues [33], detected a higher bacterial diversity than previously described in the gut of sea cucumbers. They described 37 different phyla in the gut of *A*. *japonicus*, when previously only two phyla were reported. Similar findings have been done in two sea urchin species *P*. *lividus* and *L variegatus* [42,43].

When these studies are compared to our results, some interesting findings appear. The sea urchin *L variegatus* presents an almost exclusive abundance of Proteobacteria in the gut, and of these most belong to the *Campylobacteraceae* family [43]. This decreased biodiversity can be due to a proposed compartmentalization of gut bacteria that is separated from those in the ingesta pellet as proposed by the authors or to the specialized feeding strategy of the animals that depend mainly on sea grass for their nutrition.

More interesting is the comparison with *A*. *japonicus*. The gut content of both holothurian species show a high representation of Proteobacteria. However, while in *A*. *japonicus* the phylum *Proteobacteria* was the predominant group, our results in *H*. *glaberrima* show it as being one of three main groups represented. *A*. *japonicus* did not have an abundance of the *Bacteroidetes and Firmicutes* phyla. Moreover, *A*. *japonicus* also showed an abundance of *Acidobacteria*, *Actinobacteria*, *Planctomycetes*, and *Chloroflexi* that were not present (or present lower abundance) in our study. Interestingly, both pyrosequencing studies detected a high number of unknown bacteria that could not be classified by the database, making it possible that future studies could be directed to the identification of bacteria that have not yet been discovered.

There are many important differences between the two species that might influence their microbial diversity. *H*. *glaberrima* is a tropical and semi-tropical species in the Atlantic Ocean that is suspension-feeder and a detritivore, an animal that feeds on organic matter and detritus that comes from the action of waves breaking on the rocks that serve as the animal’s habitat [62, 63]. *A japonicus* is an epibenthic deposit-feeder that ingests sediments directly from the bottom floor, mainly found in temperate climates of the northern-western Pacific Ocean [33]. Although the main food sources of both are bacteria, microalgae, meiofauna, and dead organic matter of plant and animal origin [62, 64–67] the specific environment or food availability might be key to defining their microbiota.

### Taxonomic, bacterial proportions and community structure among the three segments of the intestine of *H*. *glaberrima*

Our findings in *H*. *glaberrima* show that the distribution of microbes throughout the intestine is not homogenous. The anterior and medial intestines share a similar bacterial composition of *Proteobacteria* and *Fusobacteria* as predominant groups, while the posterior intestine (hindgut) has a higher diversity of microorganisms: *Bacteroidetes*, *Firmicutes* and *Proteobacteria* as predominant groups. The weighted PCoA plot showed that microbial communities of the anterior and medial intestines clustered together, while the posterior intestine was significantly different, this indicates similarities in the diversity and abundance of their microbial community. Our finding agreed with the results in *A*. *japonicus* that also reveal differences in bacterial communities between intestinal segments: in their case the anterior and posterior intestinal segments [33]. In the anterior gut content the most abundant phyla were *Acidobacteria*, *Actinobacteria*, *Planctomycetes*, *Chloroflexi*, and *Proteobacteria*, being the latter less abundant. On the other hand, the posterior gut content showed an abundance of *Proteobacteria*, and a low abundance of other phyla [33]. These results contrast with those obtained in *H*. *glaberrima*. As described above, the bacterial community of anterior and medial intestine of *H*. *glaberrima* showed an abundance of *Proteobacteria*. The posterior intestine (*H*. *glaberrima*) also reflected a difference in bacterial community. The most predominant groups were *Firmicutes*, *Bacteroidetes* and a low abundance of *Proteobacteria* (Fig 3). Despite these differences, at genera level we found some similarities between the two sea cucumbers: the genus *Vibrio*, the family *Desulfobulbaceae* and the class *Gammaproteobacteria* were dominant in the anterior parts of both animals. Moreover, although not abundant, both animals shared the presence of the genera *Lactobacillus* and *Vibrio* in posterior gut contents.

### Comparison of bacterial taxonomy of the digestive tract between natural and aquarium environments

Our results showed a notable bacterial difference between the holothurian intestinal microbiota obtained from a natural coastal environment and those kept in indoor aquaria. It might be suggested that these differences in bacterial composition occur due to the intake of food available within the sea cucumber’s environment. In the still waters of the aquarium environment, *H*. *glaberrima* specimens do not have the ability to feed as they do in the ocean, and their digestive tract is usually empty of the detritus, organic and inorganic matter that can be found within animals in natural conditions. (Animals can be kept in the aquarium for over 2 months. It is not certain if these “unfed” animals are obtaining nutrients from other sources, such as aquarium bacteria”. Nonetheless, “unfed” animals, serve as controls for animals that have eviscerated their digestive tract and are in the process of regeneration, since the latter lack a functional digestive tracts for at least two weeks.) Our data suggest that bacterial groups found in the anterior digestive tract of animals in natural environments but not of those in aquaria, such as *Fusobacteria* and the *Proteobacteria-Vibrio*, could be originating from the food intake. For example, it was found that differences in bacterial communities in the foregut (anterior intestine) may be caused by the selective feeding of the sea cucumber [33, 68–70]. These animals may use the bacteria directly as food source or they can use the bacteria indirectly to provide them with essential nutrients [31,32,71]. In addition, it has been suggested that the variation in the bacterial composition could be due to the food source of the sea cucumber, because it is known that the process of succession (the progressive replacement of one community by another until a climax community is established) can be caused by host external factors such as exposition to new microbes that enter the gastrointestinal tract through food [72].

### *H*. *glaberrima* core microbiota

Our finding that the posterior intestinal segment of animals in the sea environment was similar to the posterior segment of intestine of animal from the aquarium environment suggests that this segment was less susceptible to changes in its microbial composition despite changes in environment. It is known that of the gut regions of invertebrates, the most susceptible to harboring an indigenous microbiota is the posterior intestine [56,57]. Bacteria in this region have access to leftover digesta and are not competing directly with their host for uptake of digested compounds. Furthermore, the posterior intestine function is to eliminate waste material from the body [73], therefore, it is expected that the bacterial composition of this segment could help to carry out this function, after the food has been digested.

**Fig 5:**
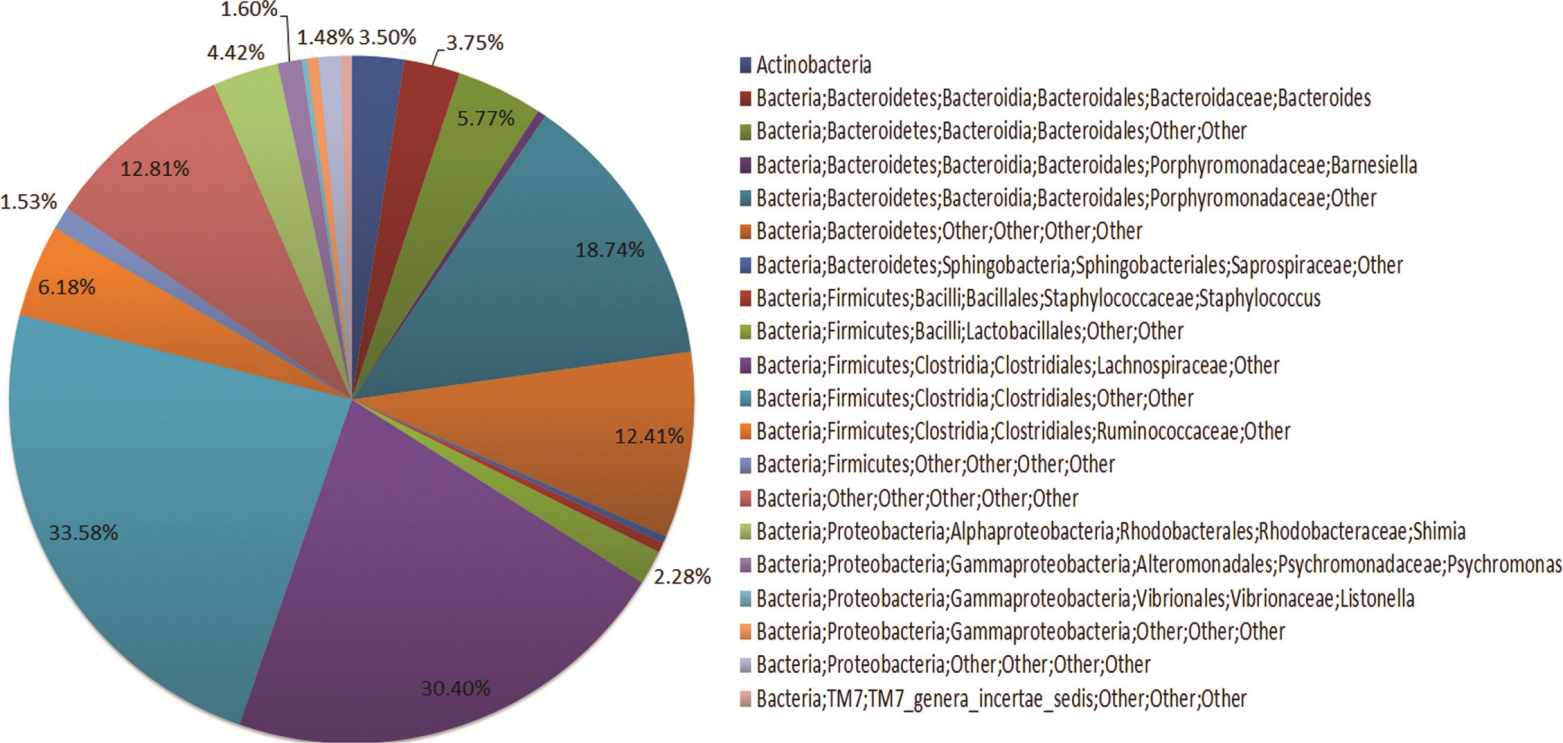
Proposed endogenous bacterial community of *H*. *glaberrima* intestine. We propose that the microbiota found in the posterior intestine of the sea cucumber, either in their natural habitat (ocean) or aquarium environments represents the endogenous microbiota of these animals.

Based on the results and analysis of the taxonomy and the study of the different environments we can propose the bacterial community found in the posterior intestine represents the intestinal core microbiota for *H*. *glaberrima* (Fig 5). The most abundant groups would be from the phylum *Bacteroidetes*: the family *Porphyromonadaceae* (18.74%), and others *Bacteroidetes* (12.41%); and from the phylum *Firmicutes*: the family *Lachnospiraceae* (30.40%), the order *Clostridiales* (33.58%) and the family *Ruminococcaceae* (6.18%). The phylum *Proteobacteria* would be less abundant, with a representation of the genera *Shimia* (4.42%), *Psychromonas* (1.60%) and *Listonella* (0.38%), the class *Gammaproteobacteria* (0.73%) and other *Proteobacteria* (1.48%).

At genera level, there are six (6) specific members of the core microbiota of *H*. *glaberrima*. These are: *Bacteroides*, *Barnesiella*, *Staphylococcus*, *Shimia*, *Psychromonas* and *Listonella*.

A particularly interesting case is the presence of *Shimia* as part of the holothurian microbiota. *Shimia* is a novel rod-shaped marine proteobacterium isolated from a biofilm in a coastal fish farm [74] and from the gut of abalone [75]. It is motile and grows on marine agar as colorless or beige colonies [74]. In our study, this genus was found in the posterior intestine sample for both environments, and these are the only regions of the sea cucumber intestine that contains *Shimia* in high proportions compared to the other segments of intestine.

The finding that the microbiota of the posterior intestinal segment is similar between animals in the natural and the aquarium environments is of importance to our future regeneration studies. It provides a baseline comparative value that can be reproduce in the laboratory and analyzed to determine the possible changes taking place during intestinal regeneration.

In conclusion, this is the first high-throughput study characterizing the microbiota of the intestine of *H*. *glaberrima*, which can be used, along with other echinoderm microbiome studies as a base for understanding the microbial ecology of these marine invertebrates. We also present here the first study that compares the bacterial composition of different segments of intestine in two different environments, which can shed a clearer view of the core microbial community of this organism, and provides important changes in the microbiota of this animal that should be taken into account when performing other studies in aquaria.
